# Editorial: Immunoendocrine system from physiology to pathology

**DOI:** 10.3389/fendo.2024.1474004

**Published:** 2024-08-22

**Authors:** Isabel Méndez

**Affiliations:** Instituto de Neurobiología, Universidad Nacional Autónoma de México, Campus UNAM-Juriquilla, Querétaro, Mexico

**Keywords:** immunoendocrine interactions, reproductive physiology, thymus function, glutamate system, cancer therapy

The intricate interaction between the endocrine and immune systems, known as the immunoendocrine system, is mediated through specific receptors that respond to environmental signals. In this Research Topic, the authors highlight the critical role of communication between these systems in both physiological and pathological contexts, with a particular focus on cancer.

In reproductive physiology, the endometrium undergoes a process called decidualization, essential for oocyte implantation. This process is influenced by hormonal signals and a pro-inflammatory environment resulting in the development of a secretory epithelium and the presence of innate lymphoid cells, including Natural killer (NK) cells. NK cells play a relevant role during the peri-implantation period and throughout the pregnancy. In this Research Topic, Fraser and Zenclussen review the current literature on the phenotype shifts and functional roles of NK cells in the endometrium and placenta at various stages of reproduction in mammals.

Several hormones traditionally recognized for their endocrine functions, have been found to also display immunomodulatory effects. These include growth hormone, somatostatin, insulin-like growth factors (IGFs) and ghrelin which are part of the somatostatin circuitry. Reis et al., in a critical review, suggest that these elements may have therapeutic potential for age-related or pathological thymus atrophy, particularly concerning intrathymic T cell development. Conversely, the hormonal and cellular microenvironment of the thymus not only orchestrate T cell-dependent immune responses against malign tumors but also influences the efficacy of anti-cancer therapies. Savino and Lepletier emphasize ongoing research into the complex interplay between the immune and endocrine systems in cancer immunity.

Glutamate, known as a neurotransmitter, also plays a key role in various metabolic pathways. Components of the glutamatergic system, such as glutamate receptors and transporters, have been extensively studied in the central nervous system and are now being explored in oncology. Changes in the expression of these components have been observed in cancers of non-neural organs, suggesting their involvement in cancer development. García-Gaytán et al. address the recent findings about the role of glutamatergic receptors and transporters in the physiopathology of cancer in peripheral organs. The authors suggest a potential therapeutic role of these components and also propose them as new biomarkers of cancer in liver and other non-neural organs.

Cancer immunotherapy has been increasingly used in recent years as a more effective and less toxic alternative to traditional treatments for neoplastic diseases. However, challenges remain, particularly concerning adverse effects associated with some immunotherapeutic tools. Olsen et al. provide a thorough review of strategies for preventing and managing immune-related adverse effects and their impact on long-term quality of life, underscoring the need to address these factors in immune-oncologic therapy.

